# Proline Dehydrogenase Regulates Redox State and Respiratory Metabolism in *Trypanosoma cruzi*

**DOI:** 10.1371/journal.pone.0069419

**Published:** 2013-07-22

**Authors:** Lisvane Silva Paes, Brian Suárez Mantilla, Flávia Menezes Zimbres, Elisabeth Mieko Furusho Pral, Patrícia Diogo de Melo, Erich B. Tahara, Alicia J. Kowaltowski, Maria Carolina Elias, Ariel Mariano Silber

**Affiliations:** 1 Departamento de Parasitologia, Instituto de Ciências Biomédicas, Universidade de São Paulo, São Paulo, Brazil; 2 Laboratório Especial de Toxinologia Aplicada (LETA) Center for Applied Toxinology (CAT/CEPID), Instituto Butantan, São Paulo, Brazil; 3 Departamento de Bioquímica, Instituto de Química, Universidade de São Paulo, São Paulo, Brazil; Louisiana State University, United States of America

## Abstract

Over the past three decades, L-proline has become recognized as an important metabolite for trypanosomatids. It is involved in a number of key processes, including energy metabolism, resistance to oxidative and nutritional stress and osmoregulation. In addition, this amino acid supports critical parasite life cycle processes by acting as an energy source, thus enabling host-cell invasion by the parasite and subsequent parasite differentiation. In this paper, we demonstrate that L*-*proline is oxidized to Δ^1^-pyrroline-5-carboxylate (P5C) by the enzyme proline dehydrogenase (TcPRODH, E.C. 1.5.99.8) localized in *Trypanosoma cruzi* mitochondria. When expressed in its active form in *Escherichia coli*, TcPRODH exhibits a K_m_ of 16.58±1.69 µM and a V_max_ of 66±2 nmol/min mg. Furthermore, we demonstrate that TcPRODH is a FAD-dependent dimeric state protein. TcPRODH mRNA and protein expression are strongly upregulated in the intracellular epimastigote, a stage which requires an external supply of proline. In addition, when *Saccharomyces cerevisiae* null mutants for this gene (PUT1) were complemented with the *TcPRODH* gene, diminished free intracellular proline levels and an enhanced sensitivity to oxidative stress in comparison to the null mutant were observed, supporting the hypothesis that free proline accumulation constitutes a defense against oxidative imbalance. Finally, we show that proline oxidation increases cytochrome *c* oxidase activity in mitochondrial vesicles. Overall, these results demonstrate that *TcPRODH* is involved in proline-dependant cytoprotection during periods of oxidative imbalance and also shed light on the participation of proline in energy metabolism, which drives critical processes of the *T. cruzi* life cycle.

## Introduction

**Figure 1:**
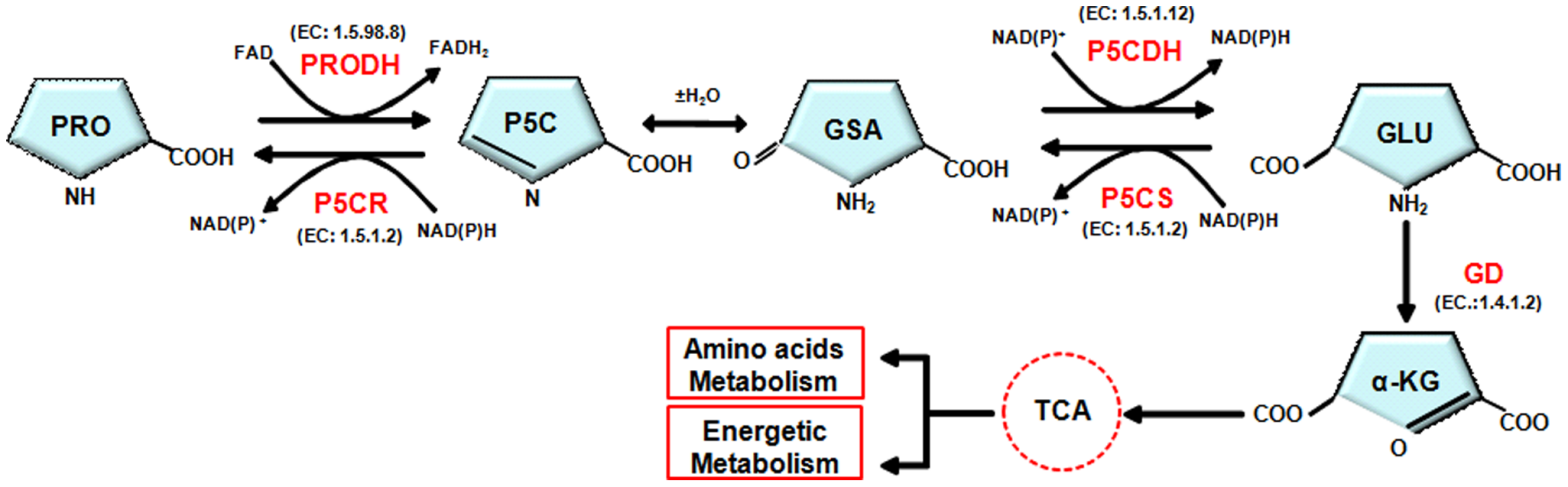
Proline metabolism. Abbreviations: PRO, proline; PRODH, proline oxidase (a.k.a. proline dehydrogenase); P5CR, pyrroline-5-carboxylate reductase; P5C, pyrroline-5-carboxylate; GSA, glutamic semialdehyde; P5CDH, pyrroline-5-carboxylate dehydrogenase; P5CS, pyrroline-5-carboxylate synthase; GLU, glutamate; α-KG, α-***ketoglutarate***; TCA, tricarboxylic acid cycle; NAD(P)^+^, oxidized nicotinamide adenine dinucleotide (phosphate); NAD(P)H, reduced nicotinamide adenine dinucleotide (phosphate); FAD, oxidized flavin adenine dinucleotide; FADH_2_, reduced flavin adenine dinucleotide.

In many organisms, proline is oxidized to glutamate through two mitochondrial enzymatic steps. The first step can be catalyzed by different two types of proline dehydrogenases (PRODH) (also known as proline oxidases – EC 1.5.1.2 and EC 1.5.99.8), which oxidize L-proline to Δ^1^-pyrroline-5-carboxylate (P5C) in either a NAD(P)^+^ or a FAD-dependent way, respectively. P5C is subsequently hydrolyzed in a non-enzymatic manner to glutamic acid gamma-semialdehyde (γSAG). In the second enzymatic step, γSAG is oxidized to L-glutamate by Δ^1^-pyrroline-5-carboxylate dehydrogenase (P5CDH), which is a NAD(P)^+^-dependent enzyme [1], [2]. Glutamate, in turn, can be deaminated by transaminases or dehydrogenases to be converted into the tricarboxylic acid (TCA) intermediary α-ketoglutarate (Figure 1). Apart from contributing to the cellular energy supply, L-proline oxidation plays an important role in intracellular redox homeostasis in a variety of organisms including fungal pathogens [3], yeast [4], [5], bacteria [6]–[8], plants [9]–[11] and mammalian cells [12], [13]. Moreover, L-proline is involved in defense mechanisms against various abiotic and biotic stresses, thus benefiting a broad range of organisms [14], [15]. However, the mechanisms of proline-mediated stress protection and, in particular, the components involved in proline-dependent signal transduction pathways are still not well understood.

*Trypanosoma cruzi,* the etiological agent of Chagas’ disease, has a complex life cycle which alternates between its insect vector, the blood-sucking “kissing bugs” of the subfamily Triatominae (Family: Reduviidae), and vertebrate hosts, including humans. *T. cruzi* epimastigotes (the predominant replicative form in the insect vector) consume glucose preferentially. After glucose exhaustion, amino acids [16] are utilized. L-proline is particularly relevant since putative genes for PRODH and P5CDH have been identified in the *T. cruzi* genome database [17] and it may be used as the main energy and carbon source [18]. Indeed, proline is involved in a variety of critical biological processes in *T. cruzi*, which are essential for life-cycle progression and pathogenesis. This includes a role in cell differentiation in the insect vector [19], [20] and in the mammalian host [21], as well as in the infection of host-cells [22]. There is also evidence of participation of this amino acid in osmoregulation [23]–[25] and resistance to nutritional and oxidative stress [26]. Furthermore, recent results have shown that proline, instead of glucose, is consumed during the intracellular life cycle stage of *T. cruzi* [27].

Despite the known involvement of the proline in several *T. cruzi* biological processes, PRODH, a key enzyme for proline metabolism has not been well characterized in this parasite. In this study, we demonstrate that the putative *TcPRODH* gene encodes a functional PRODH protein in *T. cruzi*. We have shown its involvement in the intracellular accumulation of proline and that downregulation of TcPRODH contributes to the parasite’s resistance to oxidative imbalance. Finally, we show that TcPRODH feeds electrons into the *T. cruzi* respiratory chain through the reduction of FAD.

## Materials and Methods

### Trypanosome and Yeast Cell Cultures

Epimastigotes of *T. cruzi*, CL-14, a clone derived from the CL strain [28], were maintained in exponential growth phase by subculturing every 48 hours in liver infusion-tryptose (LIT) medium supplemented with 10% (v/v) FCS at 28°C [29]. The amastigotes, intracellular epimastigotes and trypomastigote forms were obtained by infection of Chinese Hamster Ovary cell line (CHO-K_1_) cells with trypomastigotes, as described [21], [30]. Host cells were infected and maintained at 33°C, as the intracellular cycle of that strain is temperature sensitive, as described [31].Under these conditions, amastigotes and intracellular epimastigotes were purified from CHO-K1 on day 2 and around day 5 post infection respectively. Trypomastigotes are collected in the extracellular medium from seventh day on. When necessary, the intracellular forms were purified after disrupting the infected cells with a rubber policeman. After centrifugation, the pellet was suspended in RPMI medium and purified by centrifugation on 5 ml lymphoprep (Nycomed Pharma AS) for 10 min at 4300 *g*. The *Saccharomyces cerevisiae* strain YLR142W (BY4741; Mat a; his3Δ1; leu2Δ0; met15Δ0; ura3Δ0; YLR142w::kanMX4) knockout for *PUT1-* proline dehydrogenase was obtained from the EUROSCARF collection (Germany). This yeast strain was routinely cultured in YPD medium (1% (w/v) yeast extract, 2% (w/v) peptone, 2% (w/v) dextrose) containing 200 µg/ml G418 or synthetic dropout (SD) media with appropriate supplements at 30°C. When indicated, proline was added to the SD medium.

### Protein Extracts and Mitochondrial Preparation

Parasite protein extracts were obtained through three cycles of freeze-thawing in lysis buffer (50 mM Tris-HCL buffer pH 7.6, 0.25 M sucrose, 0.2% v/v Triton X-100) containing 1 µM phenylmethyl-sulphonyl fluoride (PMSF), 0.5 mM N-alpha-p-tosyl-lysyl-chloromethyl ketone (TLCK) and 0.1 mM trans-epoxysuccinyl-L-leucyl amido (4-guanidino) butane (E-64) as protease inhibitors. Yeast cells were then resuspended in buffer A (0.1 M Tris–HCl pH 7.4, 100 mM KCl, 10 mM MgCl_2,_ 0.1% v/v Tween 80, 0.1 mM EDTA and 1 µM PMSF) and ruptured with 0.5 g glass beads (0.5 mm diameter) by vortexing for five 2 min sets, with a 1 min interval between each set. Rupture of the cells was confirmed by microscopy. The samples were centrifuged at 7000×g for 10 min at 4°C. Mitochondrial fractions were obtained from log phase cultures of *T. cruzi* epimastigotes using a modified protocol of Schneider et al., (2007) [32]. The protein concentration was determined by the Bradford assay, using bovine serum albumin as a standard [33].

### Cloning, Expression and Purification of the Recombinant *Tc*prodh Protein

The putative *TcPRODH* gene (Tc00.1047053506411.30) was identified from the *T.cruzi* genome project database (http:/www.genedb.org). The *TcPRODH* coding region was amplified by PCR using *T. cruzi* genomic DNA as template and gene-specific primers designed with restriction sites for the enzymes *BamHI* and *EcoRI* (underlined): TcPRODH-Forward 5′-TTAGGATCCTCTCCAACTTCACGCAAAATTC-3′ and TcPRODH-Reverse 5′-GAATTCCTAAGCCTTTACATCTTTTTCCCG-3′. PCR amplification settings were set at 95°C (5 min) and 32 cycles using the following conditions: initial denaturation cycle at 92°C (1 min), annealing at 55°C (1 min) and elongation at 72°C (2 min). A single fragment (1.7 kb) was amplified and the PCR product was purified from a 1% (w/v) agarose gel and cloned into the pGEM-T Easy vector (Promega, Madison, WI). Selected clones were sequenced and the expected identity of the cloned DNA fragments to PRODH was confirmed by using the BLAST software program (http://blast.ncbi.nlm.nih.gov/). The gene encoding the putative PRODH enzyme was further subcloned into the pAE expression vector [34] and the construct was used to transform *E. coli BL21-*CodonPlus (DE3) cells. The bacteria were grown in Luria-Bertani (LB) medium containing 100 µg/mL ampicillin and 5 µg/mL tetracycline at 37°C, until an OD_600_ of 0.6 was reached. Expression of TcPRODH was induced by the addition of isopropyl-1-thio-β-D-galactopyranoside (IPTG) to a final concentration of 0.5 mM and cells were maintained at 37°C for 4 hours. For protein purification, the cells were harvested, resuspended in lysis buffer (50 mM Tris-HCL pH 7.5, 500 mM NaCl, 0.1% (v/v) Triton X-100 and 1 mg/mL lysozyme) containing protease inhibitors and subjected to three cycles of sonication (three 1 min pulses followed by 1 min rest between cycles) at 4°C. The recombinant 6-His-tagged protein was purified using Ni^2+^-nitrilotriacetic (NTA) column affinity chromatography (Qiagen®) according to the manufacturer’s instructions.

### Reverse Transcription PCR (RT-PCR) and Quantitative Real-Time PCR (qRT-PCR)

Total RNA was extracted from different *T. cruzi* stages, CHO-K_1_ cells (control) and yeast cells using TRIzol reagent (Invitrogen, Life Technologies). RNA preparations were treated with RNase-free DNase I (Fermentas, Life Sciences) and checked by running aliquots in 1% agarose gels. Reverse transcription was performed with SuperScript IITM (Invitrogen, USA) using the anti-sense Oligo (dT) primer, 5 µg of RNA and by following the manufacturer’s instructions. The primers used for qRT-PCR analysis were designed using software provided by Eppendorf (RealPlex v.1.5). Primers were designed based on the nucleotide sequences of *T. cruzi* glyceraldehyde-3-phosphate dehydrogenase (GAPDH) (GeneBank accession number: AI007393), which was used as a housekeeping gene [27] and *TcPRODH* (GeneBank accession number: XM800962). Primer sequences used were: GAPDH forward (5′-GTGGCAGCACCGGTAACG-3′), GAPDH reverse (5′-CAGGTCTTTCTTTTGCGAAT-3′), TcPRODH forward (5′-ACGCAAAATTCAGCCGGTAA-3′) and TcPRODH reverse (5′-GGCTCGCACTAACCACCAAA-3′). qRT-PCR analyses were performed using Mastercycler® ep Realplex 1.5 (Eppendorf, Germany) equipment and a SYBR Green QuantiMix EASY SYG KIT (Biotools Quantimix EasySyg, Spain) for amplicon quantification. PCR conditions were as follows: initial denaturation at 95°C (10 min) followed by 40 cycles of 94°C (1 min), 57°C (1 min) and 72°C (2 min). In all cases, denaturation curves for the PCR products were obtained. Data obtained were analyzed using REALPLEX v1.5 software. A fold-change in the expression of transcripts was obtained using the 2-ΔCT method [35]. All time-fold variations were calculated using GAPDH as a housekeeping protein. cDNA from CHO-K_1_ cells was used as a control, as previously described [27].

### SDS-PAGE and Native gel Electrophoresis

Sodium dodecyl sulfate polyacrylamide gel electrophoresis (SDS-PAGE) was undertaken using 10% (v/v) polyacrylamide gels, according to the method of Laemmli [36]. The approximate molecular mass of the native enzyme was determined using blue native gradient electrophoresis (BNGE) [37], [38]. Mitochondrial membranes were solubilized using dodecyl-b-D-maltoside and digitonin, and a detergent-to-protein ratio of 2∶1 and 4∶1, respectively, was employed. Samples were centrifuged at 20,000×g for 20 min at 4°C. Approximately 10 µg protein from the total mitochondrial extract was applied into each well of a 3–15% (v/v) polyacrylamide gradient gel. Protein bands were visualized by staining with Coomassie brilliant blue R-250.

### TcPRODH Activity

Two methods were used to determine the enzymatic activity of TcPRODH. The first method measured the levels of P5C formed using *o*-aminobenzaldehyde (OAB), and the resultant OAB-P5C complex was quantified by spectrophotometry at 443 nm. The assay reaction mixture (1 mL) contained 15 mM proline, 15.6 µg cytochrome *c*, 100 mM potassium phosphate buffer pH 7.5 and 50 µl of 2.5% (w/v) *o*-aminobenzaldehyde (in 40% (v/v) ethanol). The reaction was started by adding 50 µg of the recombinant protein to the assay reaction mixture, incubating for 30 min at 37°C, and stopped by adding 1 mL of 10% (v/v) trichloroacetic acid and centrifugation at 5000×g for 10 min. The clear, yellow supernatant solution was carefully removed and the absorbance was measured at 443 nm (ε = 2710 M^−1^cm^−1^). One unit of enzyme specific activity was expressed as 1 nmol of P5C formed per min/mg of protein. The blank incubation was treated in the same manner, except that L-proline was omitted.

The second method determined TcPRODH activity by measuring the reduction of the electron-accepting dye dichlorophenolindophenol (DCPIP) at 600 nm. The DCPIP reaction mixture contained 11 mM MOPS, 11 mM MgCl_2,_ 11% (v/v) glycerol, 0.28 mM phenazine methosulfate and 56 µM of DCPIP pH 7.5. Varying proline concentrations were added to between 900 and 950 µl of the stock assay mix, and the reaction (1 mL total volume) started by adding the enzyme (1–50 µl) [39]. An absorption coefficient () of 21 mM1. cm1 at 600 nm was used for DCPIP [40]. K_m_ and V_max_ values for recombinant *Tc*PRODH were determined by regression analysis of the initial reaction velocity versus proline concentration using the Michaelis-Menten equation. The concentration of flavin-bound PRODH was determined using the molar extinction coefficient for bound FAD (ε_451_ = 10,800 M^−1**.**^ cm^−1^), NAD^+^ and NADH FAD (ε_340_ = 6,220 M^−1**.**^ cm^−1^) [41]. The optimum pH for recombinant TcPRODH activity was determined using a mixed buffer system, which ranged from pH 5.0 to 11.0 and was comprised of 50 mM each of cacodylate, MOPS, MES, Tris, CAPS and CHES. The kinetic parameters of the enzymatic reactions were calculated based on data obtained from at least three independent experiments.

### Cytochrome *c* Reduction

Proline-dependent cytochrome *c* reduction was measured in a 1 ml reaction mixture which contained 0.05 M Tris-HCl pH 8.5, 5 mM MgCl_2_, 0.5 mM FAD, 0.5 mg cytochrome *c*, 0.1 M proline and the mitochondrial preparation (with or without antimicyn A). The reaction was monitored by spectrophotometric analysis at 550 nm. The concentration of reduced electron acceptors was determined using the molar extinction coefficient, which is 1.8×10^4^ for cytochrome *c*.

### TcPRODH Antisera and Western Blot Analysis

The N-YTEDRVFNDLTRSELE-C region of TcPRODH was selected to produce synthetic peptides since this region corresponds to the best combination of hydrophobicity and predicted antigenicity, according to the algorithm described by Kyte and Doolittle [42]. The synthetic peptide was coupled to keyhole limpet hemocyanin (KLH, Thermo Scientific-Pierce, USA) following the manufacturer’s instructions. Four-week-old wild-type BALB/C male mice were intraperitoneally inoculated (every 15 days) with 50 µg of the KLH-coupled synthetic peptide in Freund?s adjuvant. After five weeks, blood was collected and the serum was separated. Before immunization, a blood sample was collected as the pre-immune control. Antibodies were affinity purified by adsorption to the KLH-coupled peptide immobilized on nitrocellulose membranes, as previously described [43]. The protocols used herein were approved by the ethical committee of the Institute of Biomedical Sciences, University of São Paulo, according to national regulations and international standards for animal care.

For Western blot analysis, recombinant TcPRODH (50 µg/lane) was loaded onto a 10% (v/v) polyacrylamide gel and subsequently transferred to a nitrocellulose membrane. Membranes were blocked with 5% (w/v) non-fat milk in TBS-T (Tris-buffered saline, containing primary 0.1% v/v Tween 20) for 1 hour at 23°C. This was followed by incubation with an anti-PRODH antibody (dilution 1∶500) for 1 hour at room temperature with constant agitation. The blots were washed with TBS-T and incubated with horseradish peroxidase (HRP) coupled to an anti-mouse secondary antibody (Jackson ImmunoResearch, USA), which was diluted 1∶5000 in TBS-T. The bound antibodies were visualized using ECL Western blot detection reagents (Thermo Fisher Scientific, USA). Competition assays were performed by incubating the nitrocellulose membranes with synthetic peptide diluted in TBS before incubation with the anti-PRODH antibody as described above.

### Subcellular TcPRODH Localization

To determine the subcellular localization of TcPRODH, two complementary approaches were used: (i) digitonin extraction of intact cells and (ii) immunofluorescence microscopy. Digitonin extraction was performed using *T. cruzi* epimastigotes as previously described [44]. Epimastigotes (6.4×10^8^ cells) were resuspended in TSEB buffer (25 mM Tris-HCL buffer, pH 7.6, 0.25 M sucrose, 1 mM EDTA and 10 µM E-64) supplemented with increasing digitonin quantities (0 to 5 mg). The parasites were incubated at 25°C for 5 min and then centrifuged at 18.000×g for 2 min at room temperature. The supernatant (S) was separated immediately. The pellet (P) was then washed with TSEB and disrupted by sonication, using 4 pulses of 20 seconds each, at 60% maximum amplitude. The activity of pyruvate kinase (cytosolic), hexokinase (glycosomal) and citrate synthase (mitochondrial) were measured as markers in all supernatant (S) and pellet (P) fractions. TcPRODH activity was measured in all fractions. Equal volumes of S and P fractions were subjected to SDS-PAGE analysis under reducing conditions, blotted onto nitrocellulose membranes and probed with specific polyclonal mouse antisera raised against PRODH enzyme.

For immunofluorescence microscopy, 1×10^6^ cells/mL of log phase parasites in different life cycle stages were resuspended in culture medium without serum containing 100 nM MitoTracker (Molecular Probes, Invitrogen) and treated according to manufacturer’s instructions for mitochondrial staining. Cells were washed with PBS and fixed with 2% (v/v) paraformaldehyde in PBS for 20 min at room temperature. Fixed cells were washed three times with PBS and incubated with a 1∶500 dilution of primary mouse antibody against PRODH for 1 hour. The coverslips were rinsed three times with PBS and incubated with a 1∶2000 dilution of goat anti-mouse IgG conjugated to Alexa Fluor 488TM in blocking solution for 1 hour. After washing the coverslips three times in PBS, they were incubated with DAPI (1 µg/ml), washed again with PBS and observed under a confocal microscope. Fluorescence images were obtained using a confocal microscope (Carl Zeiss Meta System, Germany) and processed using ImageJ 1.44 software (National Institutes of Health, MD, USA).

### Yeast Strain Construction and Complementation Analysis

The coding region of the *TcPRODH* gene was excised from the pGEM-T Easy plasmid (Promega, Madison, WI, USA) and inserted into the EcoRI restriction site in the galactose-inducible yeast expression vector pYES 2.0 (URA3-Invitrogen). The yeast strain YLR142W (ΔPUT1) knockout gene PRODH was transformed with a *TcPRODH*-bearing pYES vector (TcPRODH/pYES) or with a pYES vector without an insert (control) by following an already described method [45]. Transformed cells were selected in minimal medium containing galactose (2% w/v) but lacking the base uracil at 30°C for between 2 to 4 days. Colonies obtained were tested for recovery of their growth ability on agar plates containing 0.1% (w/v) L-proline as the only nitrogen source, or on agar plates containing a mixture of amino acids except proline as a control.

### Oxidant Tolerance Tests

Early log-phase yeast cultures at OD_600_ = 0.5 were diluted to an OD_600_ of 0.05 with appropriate synthetic dropout (SD) media containing 2% galactose. Cells were then treating with either inorganic hydrogen peroxide or *T*-butyl hydroperoxide in culture medium containing L-proline (5 mM), at 30°C with vigorous shaking for 4 hours. During this period, 10 µl cell aliquots were collected each hour, spread on YPD plates and incubated at 30°C for 48 hours. Cell viability was determined by counting the number of colony-forming units (CFUs) and comparing the number of CFUs from treated and untreated cells. All experiments were repeated in triplicate at least [4], [15].

### Thiol Determination

Reduced (GSH) and oxidized glutathione (GSSG) concentrations were determined by mediated recycling assay in the presence of NADPH and DTNB [46]. GSSG was determined by subtraction of GSH from total glutathione. The final values considered for analyses were obtained from at least three independent experiments.

### Measurement of Intracellular Proline Levels

Parasites and yeast strains (1×10^8^cells/mL) were challenged with peroxides as described above, ruptured and their proteins were precipitated by the trichloroacetic acid (TCA) method following centrifugation at 10.000×g for 30 min at 4°C. Measurements of intracellular proline in the remaining supernatant solution were performed using the ninhydrin reaction (an adapted Bates protocol) [47]. In brief, 200 µl of the supernatant was incubated with 200 µl of acid ninhydrin (0.25 g ninhydrin dissolved in 6 ml glacial acetic acid and 4 ml 6 M phosphoric acid) and 200 µl of glacial acetic acid for 1 hour at 100°C. The reaction was stopped by incubation on ice and the mixture was extracted with toluene (400 µl/sample). The toluene phase was separated and the absorbance at 520 nm was recorded. A proline standard curve ranging from 0 to 500 µM proline was used to determine the proline levels of each sample, expressed in nmol/number of cells. It is worth mentioning that Bates method is specific for proline due to the fact that, in spite is well known that ninhydrin reacts with all amino acids in a sample, proline is the only one having a different absorption spectrum with an absorption peak at 520 nm [47].

### Statistical Analysis

Results were expressed as mean ± the standard error of mean (S.E.M.) values for at least three independent experiments. Non-parametric statistical analyses were performed using the one-way ANOVA test combined with Tukey’s test. Results were considered statistically significant at p<0.05.

## Results

### *In Silico* Analysis of TcPRODH

**Figure 2:**
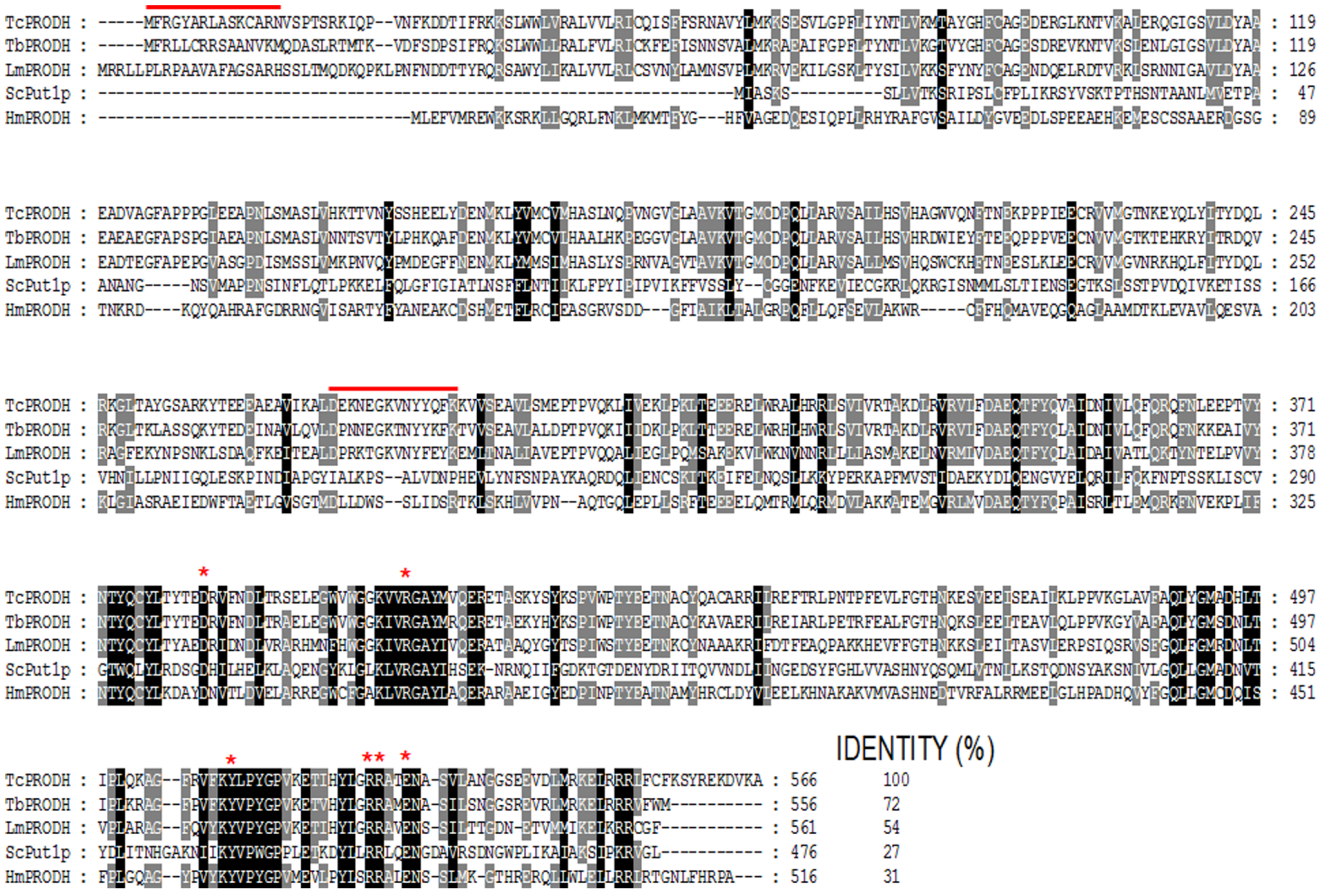
Sequence alignment of the putative TcPRODH protein (XP806055) with PRODH orthologs from other organisms. The amino acid sequence of TcPRODH was aligned with orthologs from closely related flagellated protozoa *Leishmania major* Lmj261610 (LmPRODH), *Trypanosoma brucei* Tb9277210 (TbPRODH) and eukaryotes (*Homo sapiens* AAD24775 (HmPRODH), *Sacharomyces cerevisiae* AY69290 (ScPut1p) using ClustalX default settings. Conserved (black) or substituted (grey) residues are highlighted. Degrees of identity (IDENTITY %) are shown after the C-terminal region of each sequence. The N-terminal signal peptide and putative EF-hand domain are underlined. Based on the available crystal structures of PRODH, the conserved residues implicated in cofactor (FAD binding) and substrate specificity (proline) are indicated by asterisks.

In the *T. cruzi* genome database (http://www.genedb.org/Homepage/Tcruzi) we identified an open reading frame (ORF) encoding the putative protein TcPRODH (1701 bp, Tc00.1047053506411.30), which has a predicted molecular mass of 64.7 kDa. Amino acid sequence analysis showed that, as expected, TcPRODH has the highest sequence identity with *Trypanosoma brucei* orthologs (72%), followed by orthologs from *Leishmania major* (54%), *Homo sapiens* (31%) and *Sacharomyces cerevisiae* (27%) (Figure 2). The presence of putative protein domains or conserved motifs between sequences from different species was analyzed using the InterProScan database (version 4.6) (http://www.ebi.ac.uk/Tools/pfa/iprscan/). This analysis revealed conserved regions related to the family of proline dehydrogenases, as well as a putative FAD binding domain. Additionally, a putative EF-hand motif domain (which binds Ca^2+^ and eventually other bivalent metal ions) was found, which was unexpected for this family of proteins.

Based on crystallographic data for *E. coli* PRODH (43% similarity, 26% identity to *Tc*PRODH), the key residues involved in substrate and cofactor (FAD) binding have been predicted: Asp^370^, Tyr ^540^, Arg^555^, Arg^556^, and Leu^513^ have been implicated in substrate binding, while Arg^431^ and Glu^559^ play a key role in FAD binding [48]. These residues are also conserved in TcPRODH (Asp^360^, Arg^392^, Leu^488^, Tyr^510^, Arg^525^, Arg^526^ and Glu^529^) (Figure 2). Recently, these residues have been found to be highly conserved in *S. cerevisiae* Put1 [49], which indicates a conserved catalytic mechanism between orthologous proteins from *E. coli* and eukaryotic organisms, such as trypanosomes.

Furthermore, a predicted N-terminal mitochondrial targeting signal, followed by a putative transmembrane alpha helix spanning domain, was identified (Figure 2). These data led us to hypothesize that TcPRODH is associated with the mitochondrial inner membrane in *T. cruzi*, a common feature of eukaryotic PRODH enzymes analyzed to date [39].

### Activity and Biochemical Characterization of TcPRODH

**Figure 3:**
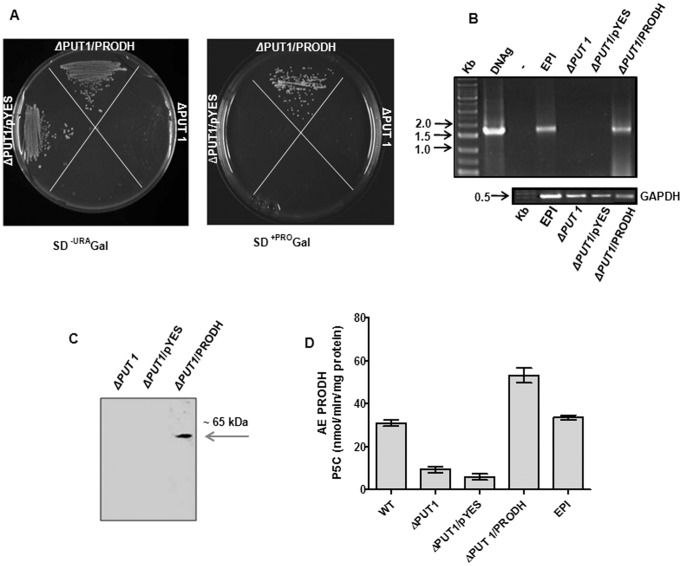
Functional complementation of a *Saccharomyces cerevisiae*Δ-*PUT1* yeast strain with the *TcPRODH* ORF (1.7 Kb). (A) The yeast strains Δ-*PUT1*, Δ-PUT1 transformed with either empty vector (Δ-PUT1/pYES) or vector carrying the PRODH gene (Δ-PUT/PRODH) were grown in culture medium containing a mix of amino acids with the exception of proline (SD^-URA^ Gal) or supplemented with proline (0.5% (v/v) as the only nitrogen source (SD^+PRO^ Gal). (B) The 1% gel shows the PCR amplified products from cDNAs obtained by reverse transcription of mRNAs of epimastigotes of *T. cruzi*, and the yeast mutant (ΔPUT1), control (ΔPUT1/pYES) and complemented (ΔPUT1/PRODH). The positive and negative controls correspond to the test reactions performed using genomic DNA from *T. cruzi* (EPI) or not (−) respectively. The GAPDH gene was used as a house keeping gene for qRT-PCR reaction (bottom panel). (C) Western blot analysis of total extracts from yeast mutant (ΔPUT1), control (ΔPUT1/pYES) and complemented (ΔPUT1/PRODH). The arrow indicates the TcPRODH band with a molecular mass of approximately 65 kDa. (D) Specific Activity of PRODH of wild-type strain BY4741 that gave rise to mutant ΔPUT1 (WT), yeast mutant (ΔPUT1), control (ΔPUT1/pYES) and complemented (ΔPUT1/PRODH), which were grown for 16 hours at 30°C in the minimum medium plus proline. Epimastigotes of *T. cruzi* (EPI) were grown in LIT medium. Enzymatic activity of PRODH within the protein extracts of the listed samples was assessed by measuring the formation of the OAB-P5C complex at 443 nm.

To verify whether the putative TcPRODH identified coded for a functional enzyme, its ability to recover the wild type phenotype of a *S. cerevisiae* PRODH null mutant (ΔPUT1p in yeasts) was analyzed. Only colonies transformed with the *pYES-PRODH* gene were able to grow in medium containing L-proline as the sole nitrogen source. Yeast cells transformed with the empty vector (ΔPUT1/pYES) and mutant strain (ΔPUT1) did not grown under these conditions (Figure 3A). This demonstrates that the product of the *PRODH* gene insert is able to oxidize proline to P5C and subsequently to glutamate, which can act as a -NH_2_ donor through the transamination network in these cells. To confirm the identity of this gene product, mRNA and protein expression levels and the activity of TcPRODH in the transformed and knockout yeasts were analyzed. A product of the size expected for the *T. cruzi* cDNA was detected in the transformed, but not in the non-transformed, yeast cells (Figure 3B). A single 65 kDa band was detected in the cells transformed with the *TcPRODH* plasmids, but not in the knockout or mock-complemented yeasts (Figure 3C). Significant PRODH activity was only observed in yeast homogenates expressing TcPRODH or wild-type (WT) *S. cerevisiae* as well as *T. cruzi* epimastigotes (control) (Figure 3D). This data confirms the PRODH activity of the cloned gene.

**Figure 4:**
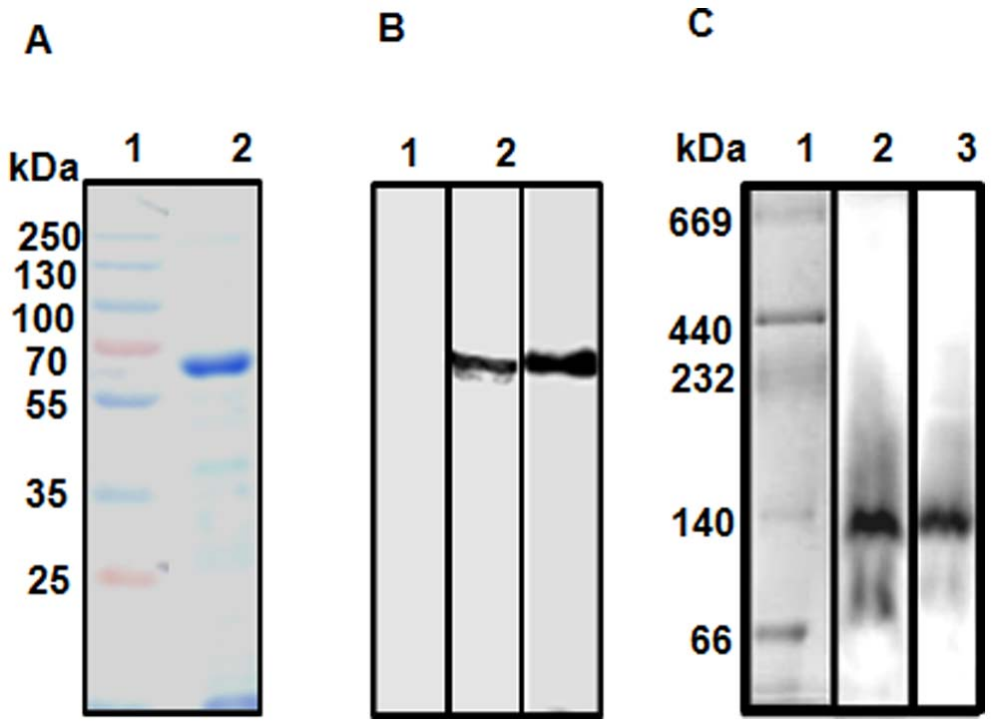
Heterologous expression and purification of recombinant TcPRODH. Each of the recombinant proteins (10 µg) was analyzed by SDS-PAGE, using 10% (v/v) polyacrylamide gels under reducing conditions and visualized by Coomassie Blue staining. (A) Lane 1: Molecular weight markers; Lane 2: recombinant TcPRODH. (B) Western blot analysis was performed using an anti-PRODH antibody raised against the recombinant enzyme. Lane 1: pre-immune serum; Lane 2: extract *T.cruzi* epimastigote stage extract; Lane 3: recombinant TcPRODH. (C) Analysis of mitochondria by Blue-Native gel electrophoresis (BNGE) of the mitochondrial preparations of different strains *T. cruzi* followed by western blot using antibody against protein PRODH (lane 1, strain CL 14, lane 2, strain CL Brenner).

To further characterize TcPRODH, the *TcPRODH* gene was subcloned into the pAE expression vector to produce the purified recombinant enzyme. Recombinant TcPRODH was expressed in *E. coli,* and purified by affinity chromatography using a Ni^2+^ NTA resin, as described in Materials and Methods. After purification, SDS-PAGE analysis revealed that recombinant TcPRODH migrated as a single band with a molecular mass of approximately 66 kDa, which corresponds to the predicted molecular mass for the 6× HIS*-*PRODH construct. Western blot analysis of the purified protein or epimastigote extracts revealed bands corresponding to the recombinant protein (66 kDa) and to the predicted protein from epimastigote lysates (65 kDa) (Figure 4A, 4B). Interestingly, Western blot analysis performed using native gels revealed a single band corresponding to a 140 kDa protein, indicating a dimeric state for native TcPRODH (Figure 4C).

**Figure 5:**
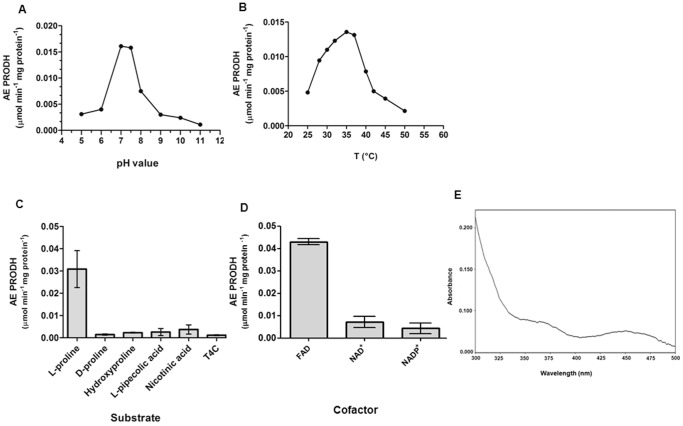
Effects of pH, temperature, substrate specificity and cofactor dependence on the specific activity of recombinant TcPRODH. Panel (A) pH dependence of TcPRODH activity. The buffers used were cacodylate and MES (pH 5.0 −6.0), TRIS buffers (pH 7.0–8.0), CHES buffers (pH 8.0–9.0) and CAPS (pH 10.0 −11.0). Panel (B) temperature dependence of TcPRODH enzyme activity. Panel (C) substrate specificity of TcPRODH. The enzyme activity was determined by using different analogs of L-proline as substrates. Panel (D) enzymatic activity was assayed using NAD^+^, NADP^+^ and FAD in optimal conditions for each case. Panel (E) UV-visible absorption peaks of purified recombinant TcPRODH. Peaks were observed at 370 nm and 450 nm, typical for the spectrum of a flavoprotein. At least three replicates were performed for each experiment. In Panel (A) and Panel (B), average values are shown. In Panel (C) and Panel (D) mean and standard error bars are shown.

To further characterize TcPRODH in terms of its substrate specificity and catalytic efficiency, the kinetic parameters of TcPRODH activity were determined using the proline:DCPIP oxidoreductase assay. The enzyme followed Michaelis-Menten kinetics for L-proline, with an apparent K_m_ 16.58±1.69 µM and a V_max_ of 66±2 nmol/min mg (Table 1). The activity the enzyme followed a bell-shaped curve, with an optimum pH between 7.0 and 7.5 using TRIS (50 mM) as the optimal buffer for reaction (Figure 5A) and the reaction exhibited its maximal activity at temperature to 37°C (Figure 5B).

**Table 1:** Kinetic constants and molecular parameters for the PRODH enzyme from *T. cruzi.*

Molecular Mass (SDS PAGE)	64.7±3 kDa
Molecular Mass (Native Gel)	∼ 140 kDa
Optimal Temperature	28°C–37°C
Activation Energy	1.207 kcal mol^−1^
Optimal pH	7.0–7.5
Recombinant PRODH	*K*m (app) L-proline 16.58±6.08 µM
	V_max_ 4.64±2.60 nmol^−1^ min^−1^ mg protein ^−1^
	V_max_/*K*m 0.280 nmol^−1^ min ^−1^ mg protein ^−1^
Epimastigote PRODH of *T. cruzi*	*K*m (app) L-proline 37.49±12.85 µM
	V_max_ 7.47±3.3 nmol^−1^ min^−1^ mg protein ^−1^
	V_max_/*K*m 0.199 nmol^−1^ min^−1^ mg protein ^−1^

The kinetic parameters which are the means of four determinations ± S.E.M., were obtained as described in methods.

The ability of TcPRODH to use alternative substrates, such as hydroxyproline, D-proline, L-pipecolic acid nicotinic acid or thiazolidine-4-carboxylate was tested. The enzyme catalyzed the dehydrogenation of L-proline, but not any of the other substrates (Figure 5C). The requirement for NAD^+^, NADP^+^ or FAD as cofactors was also analyzed. TcPRODH was found to be a FAD-dependent enzyme, since this was the only electron acceptor able to induce its activity (Figure 5D). Moreover, the purified enzyme had a typical flavoprotein absorbance spectrum, characterized by two flavin absorption peaks at 370 nm and 450 nm (Figure 5E).

### Subcellular TcPRODH Location

**Figure 6:**
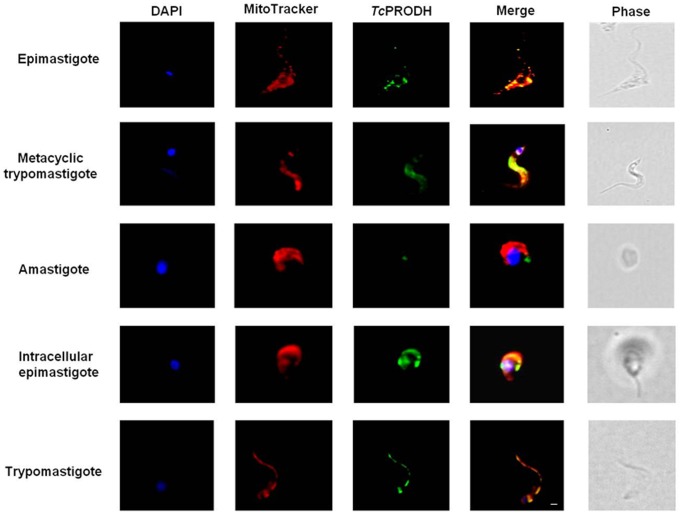
Immunofluorescence of different life cycle stages of *T.* *cruzi*. Parasites were treated with the polyclonal antibody anti-PRODH and then with a secondary antibody coupled to AlexaFluor®-455 probe (green). Parasites were labeled with DAPI for DNA staining (blue) and MitoTracker Red MitoSox (red) for mitochondrial staining. Images were merged using ImajeJ software (NIH). Phase contrast images are shown in the last column. Bars, 1 µm.

**Figure 7:**
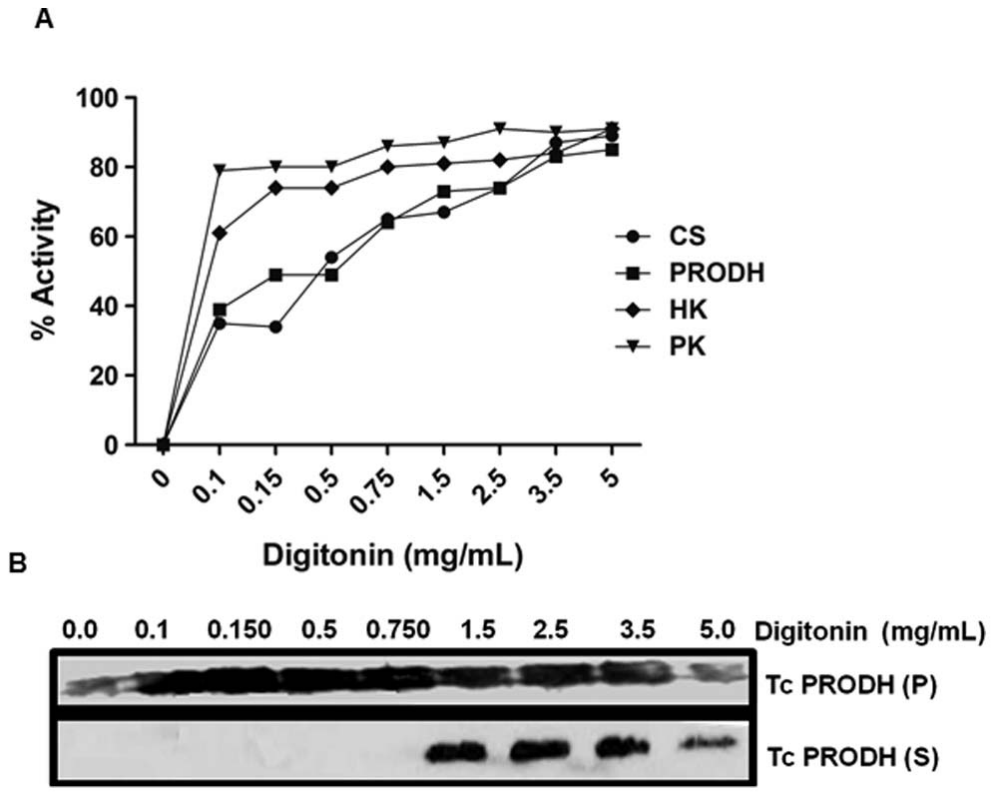
Determination of the subcellular localization of TcPRODH enzyme by digitonin extraction. Panel (A) intact epimastigotes were treated with digitonin (0–5 mg/mL) and the pyruvate kinase (PK) (inverted triangle), hexokinase (HK) (diamond), citrate synthase (CS) (circle) and proline dehydrogenase (PRODH) (square) activities were measured. (B) Soluble (S) and insoluble fractions (P) obtained at different digitonin concentrations were subjected to Western blot analysis using a PRODH specific antibody. Equal amounts of soluble and insoluble fractions were loaded per lane.

To validate the putative mitochondrial localization of PRODH as predicted by *in silico* analysis, two different assays (immunofluorescence and digitonin differential permeabilization) were performed. Immunofluorescence with anti-TcPRODH antibodies, as well as DAPI and MitoTracker staining, was performed for all stages of the *T. cruzi* life cycle. When overlapped, the signals obtained from MitoTracker and the anti-PRODH antibody indicated that, as predicted, TcPRODH is located in the mitochondrion with the exception of amastigotes, in which the fluorescence was almost undetectable (Figure 6). To confirm this result, epimastigotes were subjected to sequential permeabilization by incubation with increasing concentrations of diginonin [44]. Under these assay conditions, cytoplasmic proteins are released first, followed by glycosomal proteins and finally mitochondrial proteins. The pattern of PRODH activity release was compared to the following enzyme markers: pyruvate kinase (cytosolic), hexokinase (glycosomal) [16], [50] and citrate synthase (mitochondrial) [51]. Most of the PRODH activity (80%) was released with the mitochondrial marker citrate synthase, thus confirming the mitochondrial location of TcPRODH. This was also confirmed by immunological detection of the enzyme in the fraction corresponding to mitochondrial contents (Figure 7A, 7B).

Finally, Western blot analysis of TcPRODH expression and activity in purified mitochondrial vesicles (i.e. broken mitochondrial vesicles that had lost all of their matrix contents and were then resealed) from the epimastigote stage revealed a single band with an apparent molecular mass of 140 kDa (Figure 4C). These results confirm TcPRODH as a mitochondrial membrane located, FAD-dependent proline dehydrogenase.

### TcPRODH Expression in Different *T. Cruzi* Life Cycle Stages

**Figure 8:**
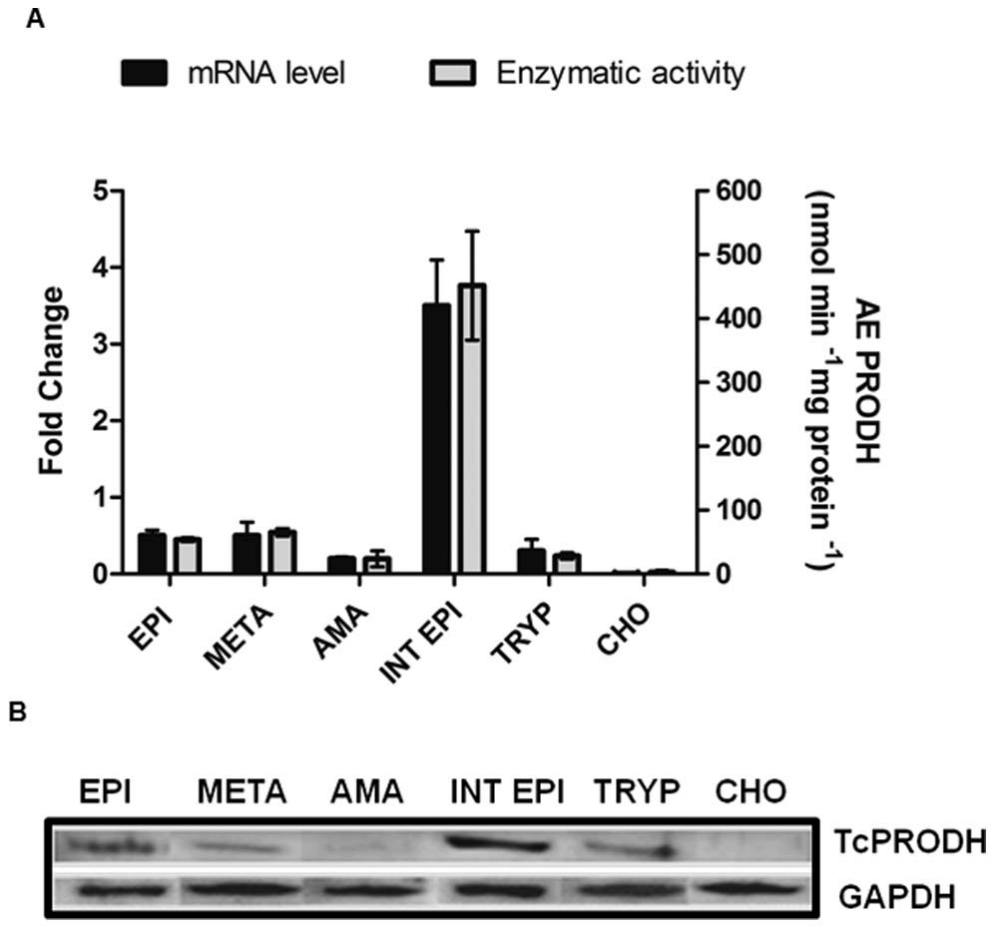
Analysis of *TcPRODH* transcript and protein abundance in different developmental stages of *T.* *cruzi*. (A) qRT-PCR analysis and specific activity protein (B) Western blot analysis of *TcPRODH* expression in different developmental stages of *T. cruzi* and CHO-K1 host cells (control). Epimastigote (EPI), metacyclic (META), amastigote (AMA), intracellular epimastigote (INT EPI), culture trypomastigote (TRYP), CHO-K_1_ host cell (CHO).

Previous evidence has shown that proline uptake is regulated during the *T. cruzi* life cycle, uptake by intracellular epimastigotes falls to less than 10% in culture-derived trypomastigotes, the stage which is infective to mammalian hosts [21]. This variation in proline uptake could be accounted for by regulation of the whole proline degradation pathway. To confirm this possibility, the specific activity of the enzyme was measured in all *T. cruzi* life cycle stages. The proliferative intracellular epimastigote stage exhibited the highest TcPRODH activity levels, while all other stages presented similar TcPRODH activity levels (Figure 8A). In order to investigate the regulation of TcPRODH activity during different life cycle stages, mRNA and protein levels were assessed. Western blot and qRT-PCR analysis indicate that *TcPRODH* mRNA expression levels correlate with enzyme activity levels during the *T. cruzi* life cycle. Expression of TcPRODH was found to be four-fold higher in the intracellular epimastigote form relative to the amastigote form (Figure 8A). Other *T. cruzi* life cycle stages had similar levels of PRODH transcript expression. TcPRODH transcript expression was found to be parasite-specific, as no amplification signal was detected when total RNA isolated from the host cell was used as template for the reaction (negative control). When TcPRODH protein expression levels of different forms of *T. cruzi* were analyzed, major differences in PRODH protein expression were observed in the intracellular epimastigote stage compared to the other parasite life cycle stages, as expected (Figure 8B). No band was detected in the negative control (CHO-K_1_ cells). These results suggest that TcPRODH activity is regulated with previously characterized proline uptake activities [27].

### Proline Accumulation and Oxidative Imbalance

**Figure 9:**
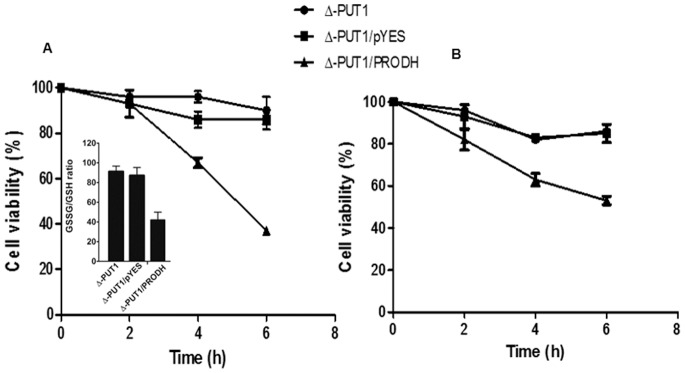
Viability of the yeast cells under conditions of oxidant challenges. Yeast strains (mutant (PUT1), control (PUT1/pYES) and complemented (PUT1/PRODH)) were cultured in the presence of (A) H_2_O_2_ or (B) t-butyl hydroperoxide for 6 hours. Cellular viability was assessed by counting number CFUs. Mean / S.E.M. values are shown. Insert (A) GSSG/GSH ratios.

PRODH has previously been reported to participate in redox regulation of several cells. Furthermore, our group demonstrated the participation of proline metabolism in the resistance to oxidative stress in *T. cruz*i [26]. Thus, it was hypothesized that TcPRODH could have an impact on redox status and resistance to oxidants in *T. cruzi* [52]. The stress tolerance levels in yeast cultures expressing TcPRODH were examined after treatment with H_2_O_2_ (hydrogen peroxide) or *T*-butyl hydroperoxide (organic peroxide). Interestingly, ΔPUT1/PRODH expressing yeasts were significantly more sensitive to both challenges (viabilities of 37% and 53% respectively) when compared to controls ΔPUT1 strain (knockout), or ΔPUT1/pYES (mock-complemented) yeast (Figure 9A, 9B). These results indicate that the presence of the functional proline oxidation pathway makes cells more sensitive to oxidants. Interestingly, these facts concur with previous results, which show that, in *T. cruzi* epimastigotes, the ability to resist H_2_O_2_ stress is related to the ability of the cells to accumulate intracellular free proline [26].To confirm the hypothesis that resistance to oxidative imbalance could be related to the free proline levels, free proline levels were measured in the three yeast strains used for the oxidative challenge experiments (i.e. the knock out, the mock complemented and the complemented strains). As expected, ΔPUT1/PRODH had the lowest levels of free proline (250±0.02 nmol/10^8^ cells). The PUT1 knockout and control (mock complemented) strains accumulated the highest levels of free proline (680±0.06 nmol/10^8^ cells and 672±0.06 nmol/10^8^ cells, respectively). This correlated with their resistance to the oxidative challenge. To confirm the role of the intracellular free proline on the redox state of the cells, the intracellular GSH/GSSG ratio was also measured after H_2_O_2_ treatment. The GSH/GSSG ratio decreased (corresponding to a more oxidized intracellular environment) in cells with a diminished proline concentration and that showed a higher sensitivity to H_2_O_2_ (Figure 9A). This finding supports previously published evidence which shows that the accumulation of intracellular free proline constitutes a mechanism contributing toward the resistance to oxidative imbalance [6], [13], [26], [53].

### TcPRODH and the *T. Cruzi* Respiratory Chain

**Figure 10:**
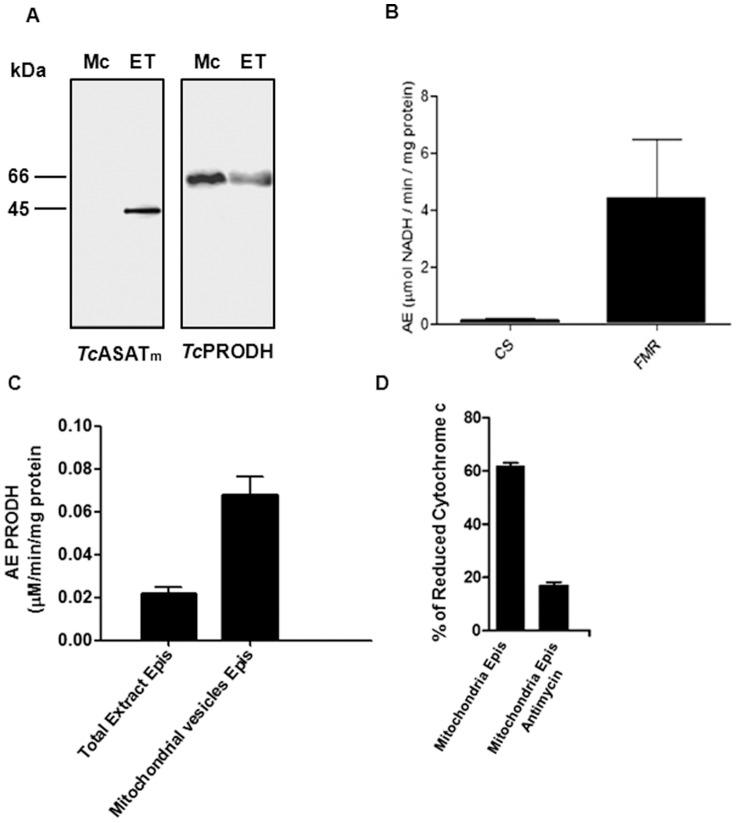
*Tc*PRODH activities in mitochondrial vesicles (Mc) and total extracts from epimastigote forms (ET). (A) Western blot analysis of ASATm (mitochondrial isoform of aspartate aminotransferase) (left panel) and TcPRODH (right panel) expression. ASATm (mitochondrial isoform of aspartate aminotransferase), an enzyme localized in the mitochondrial matrix is absent from mitochondrial vesicles. (B) Activities of citrate syntase, cs (matrix marker) and fumarate reductase, FMR (mitochondrial membrane marker). (C) Activity of TcPRODH in total extracts and mitochondrial vesicles of *T. cruzi* epimastigotes. These activities indicate that vesicles lack matrix content. (D) Reduction of cytochrome *c* in *T. cruzi* epimastigote mitochondrial vesicles in the presence L-proline, with or without antimycin A.

In most trypanosomatids, electrons can be provided to the respiratory chain by FAD- and NAD^+^-dependent enzymes. As TcPRODH the enzyme should be associated to the mitochondrial membrane, and reduces FAD, it was suggested that this enzyme could be participating in the respiratory chain by donating electrons from proline. Thus, purified mitochondrial vesicles (emptied of matrix contents) were obtained to evaluate whether TcPRODH activity was associated with the mitochondrial vesicle membranes and it participated in the transfer of electrons to cytochrome *c*. Both mitochondrial isoforms of aspartate aminotransferases (ASATm) and TcPRODH were detected, confirming again the membrane location of TcPRODH (Figure 10A). As expected, no specific citrate synthase activity (mitochondrial matrix marker) was found in the mitochondrial vesicles when compared to fumarate dehydrogenase specific activity (mitochondrial membrane marker) confirming that the vesicles were enriched with membrane-bound proteins (Figure 10B). In addition, when PRODH activity was evaluated, an activity pattern similar to that of fumarate dehydrogenase was uncovered (Figure 10C).

As previously shown, purified recombinant or native TcPRODH is able to reduce tetrazolium dyes (DCPIP). However, if TcPRODH reduces FAD, it is expected to reduce downstream cytochrome *c* [54], [55]. The question arose whether cytochrome *c* could be the natural electron acceptor in the mitochondrial membrane. Mitochondrial vesicles were prepared, and TcPRODH activity was measured over time by following the cytochrome *c* reduction by measuring the absorbance at 550 nm [56]. Upon proline addition, an oscillatory increase in the absorbance occurred, corresponding to the reduction of cytochrome *c*. This reduction was inhibited by addition of antimycin A (a mitochondrial complex III inhibitor), confirming that the observed activity was due to the transfer of electrons through mitochondrial complex III to cytochrome *c* (Figure 10D). Interestingly, under the described conditions, the cytochrome *c* reductive process occurred in regular pulses, as previously described for other metabolic pathways (data not shown). Since this oscillatory pattern is absent in the presence of unlimited quantities of electron acceptors (DCPIP), it appears that it is dependent on the dynamics of reduction and re-oxidation of cytochrome *c*.

## Discussion

This paper demonstrates that the product of the *TcPRODH* gene is a FAD-dependent L-proline oxidoreductase, the first enzyme that catabolizes proline in *T. cruzi* [57], [58]. The specificity of TcPRODH was also evaluated and closely related analogues such as D-proline, hydroxyproline, L-pipecolic acid, nicotinic acid and thiazolidine-4-carboxylic acid (T4C), did not act as enzyme substrates. Recently, Ostrander et al. [59] provided evidence that the Tyr^540^ residue in *E. coli* PutA/PRODH imposes spatial constraints in the active site, which determines the substrate preference for proline over hydroxyproline. TcPRODH is predicted to share the corresponding Tyr residue (Tyr^510^ in TcPRODH). Besides Tyr^540^, other amino acids (Asp^370^and Leu^513^) have been identified as responsible for binding the substrate and are also present in TcPRODH, as well as other organisms. Accordingly, TcPRODH showed a strict preference for L-proline and not other substrates tested, which was consistent with the presence of the Tyr^540^ residue in the active site.

The N-terminal of TcPRODH presents characteristics of a canonical mitochondrial-targeting signal peptide [60]. Indeed, we found by both immunofluorescence and differential permeabilization that the subcellular localization of the enzyme is mitochondrial, as shown in other organisms [61]. Native gel analysis of the enzyme indicates a homodimeric arrangement, which is compatible with crystallographic studies of orthologs in several cells such as *E. coli* [48], *Thermus thermophilus* [54], and *S. cerevisiae* [49].

Proline uptake in *T. cruzi* is mediated by two active transport systems; one is a high affinity, low capacity transporter called system A (K_m_ 0.35 mM), and the second is a low affinity, high capacity transporter called system B (K_m_ 1.36 mM) [62]. Intracellular free proline content, proline uptake by both transporters and glucose uptake during the mammalian host-cell infection process have been analyzed previously. Proline uptake has been demonstrated to be essential during replication and/or differentiation of the intracellular epimastigote stage into trypomastigotes [21], [27]. Accordingly, this stage exhibits the highest level of proline uptake, which is provided by the proline pool of the host-cell (approximately 0.3 mM) [62]. In this study, TcPRODH expression throughout the life cycle of *T. cruzi* was analyzed. A correlation was observed between *TcPRODH* mRNA levels, TcPRODH protein levels and TcPRODH activity in *T. cruzi* parasites. These levels increased in the intracellular epimastigotes and were diminished to the minimal levels of expression in the amastigote stage. Trypomastigotes, epimastigotes and metacyclic trypomastigotes did not exhibit noticeable differences between them. In conclusion, *TcPRODH* expression and activity is consistent with the proline uptake along the host-cell infection, supporting the hypothesis that the replicative stage intracellular epimastigotes are metabolically dependent on this amino acid.

Since proline is involved in several biological processes, apart from bioenergetics and protein synthesis [52], [63], [64], interest in its metabolism has grown. In particular, a correlation between a high intracellular free proline concentration and an increased protection from oxidative and/or thermal stresses has been described previously in bacteria, plants and yeasts [4], [5], [15], [65]. In *T. cruzi*, reduction of intracellular free proline levels make these parasites more sensitive to oxidative imbalance, thus supporting the hypothesis that accumulation of proline could contribute to resistance to oxidants [26]. To date, mechanisms involved in the process of stress protection have not been well characterized. Accumulation of free proline is controlled by a balance between uptake from the extracellular medium, biosynthesis and degradation. In PUT1 knockout yeast cells transfected with pYES/TcPRODH, decreased cell viability was found when compared with the knockout (ΔPUT1) and control (ΔPUT1?pYES) strains, thus demonstrating that, in yeast, resistance to oxidative stress is strongly linked to PRODH activity. In addition, free proline content increased in the knockout or mock-complemented cells, as the presence of the enzyme caused a decrease in the concentration of intracellular free proline. As proposed, proline accumulation may decrease intracellular levels of reactive oxygen species (ROS), thus preventing programmed cell death in fungi [3] and in mammalian cells [13]. Moreover, other studies suggest that proline acts as a potential ROS scavenger [66], [67]. However, it has also been shown that upregulation of the mitochondrial PRODH enzyme in mammalian cells leads to an increase in proline catabolic fluxes, which cause an increase in ROS production and eventual cellular death by apoptosis [68]. In addition, *in vitro* translated and purified monofunctional POX from *Thermus thermophilus* can produce superoxide from proline [54]. Thus, proline metabolism can contribute not only to bioenergetic demands and/or regulatory mechanisms, but also affect the overall intracellular redox environment.

The contribution of L-proline to cell bioenergetics has also been investigated. L-proline can power ATP production in several trypanosomatid species. When procyclic forms of *T. brucei* are cultured in a glucose poor and L-proline rich medium, oxidative phosphorylation is the preferred pathway to obtain ATP [39], [69]. *T. cruzi* epimastigotes oxidize proline to CO_2_ and H_2_O [18], which indicates that they possess an active respiratory chain which can receive electrons from proline. Previously, Martins et al. [22] have showed that metacyclic forms depleted of ATP under nutritional stress conditions were able to recover normal ATP concentrations and full infectivity when L-proline was provided. This reinforces the idea that oxidation of L-proline is able to yield ATP in *T. cruzi.* As previously established, L-proline is oxidized to glutamate, which enters the Krebs cycle at the level of α-keto glutarate to participate in energetic metabolism through the generation of succinate [70]. However, the fate of the electrons donated by L-proline oxidation remains unresolved. In *S. cerevisiae*, the oxidation of proline, via PRODH, is directly linked to the reduction of ubiquinone via complex III in the electron transport chain [49], as in bacteria, plants and animals. In these organisms, different dehydrogenases (including PRODH) transfer electrons to ubiquinone, stimulating oxygen consumption and reduction of cytochrome *c* [71]. The results presented herein show that cytochrome *c* could be an electron acceptor. Our data shows similar O_2_ consumption by mitochondrial vesicles when stimulated with either proline or succinate (data not shown). In addition, the fact that both enzymes channel electrons via FAD reduction strongly suggests that electrons coming from proline or succinate are channeled to the electron transport chain through Coenzyme Q and go downstream through an identical route. Notably, these vesicles are empty of matrix enzymes, so the proline oxidation followed by electron transfer to the respiratory chain cannot be attributed to its processing through the citric acid cycle. In summary, as with succinate, it appears that the electrons are transferred from proline via FAD, which is reduced to FADH_2_. Electrons are then transferred from FADH_2_ to coenzyme Q, through the common path i.e. complex III, cytochrome *c*, complex IV and oxygen [72].

These results suggest a key role of proline in metabolic pathways of *T. cruzi*, not only as a supplier of citric acid cycle intermediates but also as a main contributor of electrons to the respiratory chain. In addition, this study provides further support for the involvement of intracellular free proline accumulation in the resistance to the oxidative imbalance. As a whole, these results introduce an apparent conflict with respect to the role of proline: on one side, proline is a metabolite which, when accumulated, contributes toward resistances toward oxidants; on the other side, its accumulation could be contributing to ROS production due to an overload of the respiratory chain. The existence of a family of *T. cruzi* proline racemases [73], [74] allows us to propose a solution for this controversy: D-proline could be preferentially accumulated as a scavenger against oxidative imbalance. If this is the case, the relationship between PRODH and proline racemases could control the delicate balance between generation of energy, ROS production and defense against ROS accumulation. Further experimental work is required to elucidate fully the intriguing dual role of this metabolite.
